# Impact of outpatient radiotherapy on direct non-medical cost in patients in the Central Macro Region of Peru 2021

**DOI:** 10.3332/ecancer.2023.1580

**Published:** 2023-07-20

**Authors:** José Fernando Robles Díaz

**Affiliations:** Regional Institute for Neoplastic Diseases, Central Region, Concepción, Junín 12731, Peru and Universidad Peruana Los Andes, Huancayo, Junín 12731, Perú

**Keywords:** financial support, financial policy, healthcare financing, costs and cost analysis

## Abstract

**Background:**

Financial toxicity arises in cancer patients due to the objective financial burden of the disease or treatment, being associated with worse clinical outcomes. Direct non-medical spending on cancer patients undergoing radiotherapy in Peru under its publicly funded health system has not been described.

**Objective:**

To know the expenses related to the transfer of the radiotherapy outpatient.

**Methodology:**

For patients who started radiation therapy in 2021, treatment demographics and expenses related to transporting the patient from home to the radiation therapy center were prospectively collected. Association and connection tests were used, such as the Mann–Whitney/Kruskal–Wallis *U*-test and Spearman’s Rho. A value of *p* < 0.05 is considered statistically significant.

**Results:**

398 patients were collected, with average weekly expenses for transportation, lodging and food of $17.04, $6.69 and $45.91, respectively. Confirmation was positive between weekly spending and remoteness, likewise it was negative between effective teletherapy and remoteness, both analyses being statistically significant.

**Conclusion:**

The expense associated with transfer for radiotherapy is high, exceeding the average monthly income of the patient, as a consequence they have a worse therapeutic result, and may cause financial toxicity in cancer patients.

## Introduction

There is increasing recognition by oncologists of ‘financial toxicity’, a comprehensive term for harm to the patient due to the direct and indirect cost of cancer treatment [1], being recognised as an uncomfortable truth for oncologists with respect to causing severe financial stress [2].

Most studies usually consider the ‘cost’ of treatment from the perspective of what is charged to an insurer or national payer, often compared to the estimated and quality-adjusted improvements in life expectancy as a result of such an intervention; such studies are valuable and most frequently used to develop health policies. However, financial toxicity reflects financial stress at the *patient level* and not necessarily at the *institutional* or *system level.* In other words, an expensive intervention from the perspective of the payer may or may not cause financial toxicity, being an underestimated component of survival [3]. Specific out-of-pocket costs for treatment for patients who undergo outpatient radiotherapy have not been documented nationally to date and there are few publications at the Latin American level, so this study has been carried out with the objective of discovering the out-of-pocket costs related to transfer to the radiotherapy centre and if it is correlated with demographic or social factors.

## Materials and methods

In 2021, for patients who received external radiotherapy and brachytherapy at the Institute located in the Junín region of the Central Macro Region of Peru, information was prospectively recorded, obtaining the clinical characteristics with the demographic ones, through the clinical history, while the costs related to the transfer were collected through a questionnaire.

Within the inclusion criteria were the following: Patients older than 18 years old, with diagnosis of neoplasia confirmed by histology, received the first teletherapy in 2021 and more than 70% of the total teletherapy received was in the facilities. While the exclusion criteria were: Treatment regimen for disease in a state of progression, never started teletherapy for medical reasons and more than 20% of the teletherapy sessions received were in hospitalised condition.

### Patient’s personal characteristics

With respect to the insurance status, there were two options: whether the patient was cared for under Comprehensive Health Insurance [SIS (Seguro integral de Salud)] or was cared for under the ESSALUD (Seguro Social de Salud/ Social Health Insurance)/private model. The type of neoplasm was gathered from the biopsy result, while the distance data were obtained from the exact location of the home of origin, calculating the distance with the help of Google Maps software. The worst treatment outcome variable was determined as detection through imaging of progression of the disease or no changes in the irradiated tumour, during a minimum period of 12 months.

### Estimated cost

The costs associated with the transfer of the patient from their place of origin to the facilities of the institute were collected daily, specifying the cost of transportation, lodging and food. It was preferred to carry out the analysis with the weekly average rather than the total, since the pathologies had curative regimens from 1 to 10 weeks.

### Statistical analysis

All data and statistical analyses were conducted using SPSS (Statistical Package for Social Sciences, Version 28.0, Chicago, IL, USA). The descriptive statistics were performed as means or proportions. Variables were analysed using the Kolmogorov–Smirnov normality test, resulting in all variables lacking a normal distribution, forcing the use of non-parametric tests for association and correlation such as the Mann–Whitney/Kruskal–Wallis *U*-test and Spearman’s Rho, respectively.

## Results

**Figure 1.**
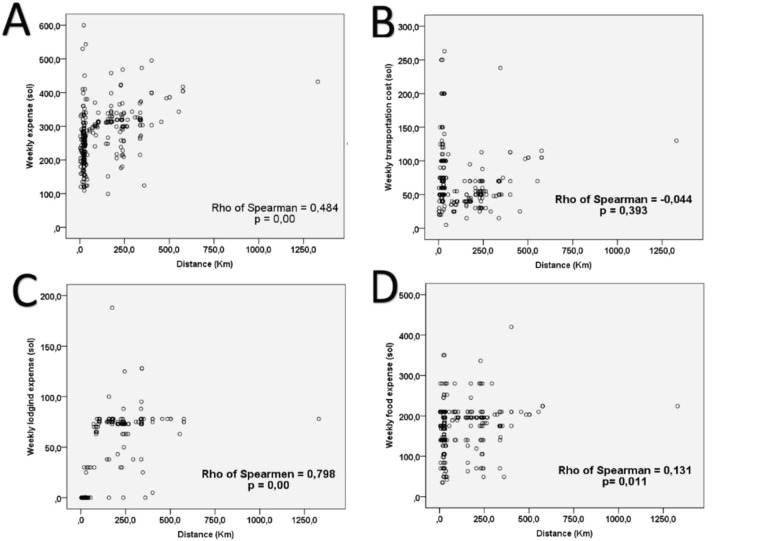
Costs associated with outpatient irradiation treatment.

**Figure 2.**
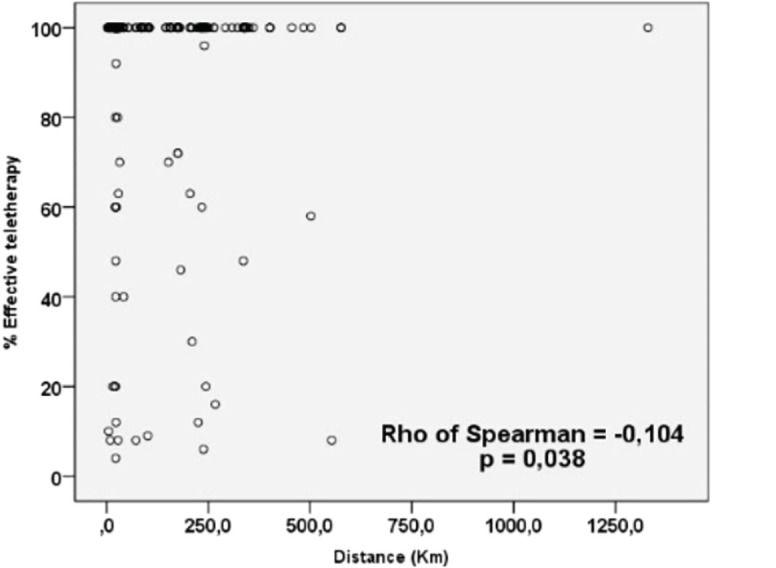
Relationship between radiotherapy regimen completed and distance.

Applying the eligibility criteria resulted in 398 patients. The characteristics of the patients, of whom only 374 started teletherapy treatment, are presented in Table 1; the distance distribution for distances less than 30, 31–150, 151–300 km and greater than or equal to 301 km was 59.29%, 11.06%, 19.60% and 10.05%, respectively. While the distribution of weekly average cost of transportation, lodging and food was $17.04, $6.69 and $45.91. The independent and dependent variables were subjected to association tests, resulting in statistical significance only for the type of insurance with the start time, while the type of neoplasm was not significant. Additionally, the correlation between distance and the worst treatment outcome is striking, either as disease progression or no response to irradiation (Table 2). There was also shown to be statistical significance between weekly cost and distance; when the costs were broken down, the correlations between lodging and food costs were significant, with good and low correlation, respectively (Table 2 and Figure 1). The low correlation between teletherapy completion rate and distance was statistically significant (Figure 2).

## Discussion

Cancer patients may be exposed to financial toxicity differently according to the country, since medical care systems are diverse [4, 5]. In our country, the direct medical cost is subsidised by ESSALUD or SIS, if the person is formally employed or unemployed/informally employed, respectively. There is a small proportion where the user can be cared for privately assuming all the costs [6, 7].

The scope of financial toxicity includes direct costs, indirect costs, patient-specific values, expectations of possible financial burdens and financial circumstances [8–10]. There are also numerous non-medical costs, including travel expenses, parking and additional family care expenses, among others, which are not currently subsidised [11].

Financial toxicity is common among patients undergoing radiotherapy; according to Fabian *et al* [4] greater subjective financial distress was significantly associated with active employment, lower quality of life, lower family income, higher direct costs and greater income loss. In our results as an institution that belongs to the state through the Ministry of Health, there is a predominance of SIS patients, and in addition, the factors that contribute the most to the direct non-medical cost are food and transportation, noting the last factor where more than 40% of the patients have to travel more than 30 km in order to receive radiotherapy care (Table 1).

Ramsey *et al* [12] found that cancer patients who filed for bankruptcy had a significantly higher adjusted mortality rate of 1.79. Additionally, there is evidence that cancer patients face financial toxicity in publicly funded healthcare systems as well [13], as shown in our study.

Regarding the weekly transfer cost generated by the outpatient treatment of the patient undergoing radiotherapy, it was $69.65 on average. The cost generated must be evaluated on a weekly basis, since the radiotherapy regimens are diverse, being identified from 1 to 8 weeks. The cost generated weekly by radiotherapy is worrisome since it represents 23.3% of the average monthly income from work in the Junín region of $298.86 [14]. If we consider this expense projected to 4 weeks, it would represent more than 90% of the average monthly income, being a risk factor for the patient to have material and psychological consequences due to financial effects.

From daily practice in the field, it is known that patients with limited financial resources who live at distances greater than 100 km often have difficulties beginning on the scheduled date, since they must save money in order to move into accommodation near to the institution. This is reflected in the results where there is a correlation between distance, with weekly expenditure and the start time for SIS patients, who represent the population with lower financial income, and it also shows consequent poor treatment outcomes with greater distance (Table 2). Given that travel distance is a known barrier to radiotherapy [15], the COVID pandemic provided an additional impetus in developed countries to improve patient-centred care by co-ordinating access to radiotherapy closer to home or in less endemic regions, since delays in care can lead to worse outcomes [16] and could be mitigated through the establishment of an accredited referral network of community practice physicians who offer high-quality radiotherapy [17]. Likewise, Cosway *et al* [18] in a sample in the United Kingdom, where patients had to travel 78.8 miles in order to receive intensity-modulated radiation therapy, considered the travel distance as the main reason for not travelling to receive treatment via that modality, and limiting themselves to only 22.7 miles in order to receive it via the conformal technique. A structural deficit exists in our country since there are only 30 linear accelerators, of which only 12 belong to public institutions [19], for 32 million inhabitants, limiting access to the service.

In practice, patients experiencing financial difficulties are less likely to adhere to prescribed treatments and follow-up, which could result in higher rates of recurrence and death from cancer [2, 20]. Patients are also increasingly worried about the financial impact of cancer and wish to discuss it, but because these discussions are not yet standardised, many feel that the current level of involvement of physicians is inadequate [9]. This is consistent with our patients, since as the distance reaches above 40 km, there is a consequent increase in weekly expenditure for the rental of housing and transportation, which increases markedly as the distance rises above 150 km, and there is a higher probability of not completing the irradiation regimen (Figures 1 and 2), being reflected in the 6% of our patients who did not start because of non-medical reasons, and for those who started radiotherapy the average completion rate was only 94% (Table 1).

Moelle *et al* [21] published the reasons for the interruption of a radiotherapy scheme, reporting 32 interruptions in 159 cases; 22% did not return for radiotherapy because of financial or logistics reasons. With regard to adherence, patients had lower chances of survival in case of discontinuation. This is consistent with our results, where there is a negative correlation between distance and level of completion of the regimen (Figure 2).

It is recommended that advantages should be taken of hypo-fractionated regimens that are just as or more effective than conventional ones. There are many studies that show the benefits of hypo-fractionation in lung cancer [22], prostate cancer [23], breast cancer [24], advanced head cancer [25], inoperable locally advanced oesophageal cancer [26] and rectal cancer [27, 28], it being as effective as conventional radiotherapy.

With regard to limitations, there was the lack of a Latin American scientific background, in relation to low output at a national level. However, our data can be extrapolated to other hospitals. The analysis has a limited number of patients who received radiotherapy, so the results will not be generalisable. Likewise, the cost evaluation is more than that expressed in the current work. In spite of this, these may be the main ones for Peru. The follow-up of patients is too short to determine a good correlation with disease progression. However, our study is one of the few that have prospectively evaluated the costs related to the transfer of cancer patients undergoing radiotherapy in a Latin American centre.

## Conclusion

Our results show that the cost associated with transfer is high, exceeding the average monthly income of the patient, and may be causing financial toxicity in cancer patients undergoing radiotherapy in Peru. Distance correlates positively with higher average weekly cost and start time, and negatively with completion of the radiotherapy regimen and the worst outcome in the first year post irradiation.

Confirmatory studies are needed to establish risk factors. Additionally, future studies should focus on prevention and interventions to improve radiotherapy satisfaction and outcomes in patients.

## Conflicts of interest

There are no potential conflicts of interest in this research.

## Funding

No funding has been received.

## Figures and Tables

**Table 1.** Clinical, demographic and financial characteristics of patients.

Variable		*N*	%	Average	Standard devn.	Normality *p*-value ^*^
Type of insurance						0.00
	SIS	272	68.34	-	-	-
	ESSALUD or private	126	31.66	-	-	-
	Total	398	100.00	-	-	-
Distance						0.00
	0–20 km	59	14.82	-	-	-
	21–30 km	177	44.47	-	-	-
	31–150 km	44	11.06	-	-	-
	151–300 km	78	19.60	-	-	-
	≥301 km	40	10.05	-	-	-
	Total	398	100.00	-	-	-
Type of neoplasm						0.00
	Cervix	166	41.71	-	-	-
	Breast	54	13.57	-	-	-
	Rectum	19	4.77	-	-	-
	Other	159	39.95	-	-	-
	Total	398	100.00	-	-	-
Weekly cost						0.00
	Transportation	-	24.47	64.76 (17.04)^a^	36.83 (9.70)^a^	-
	Lodging	-	9.61	25.44 (6.69)^a^	36.32 (9.56)^a^	-
	Food	-	65.92	174.48 (45.91)^a^	51 (13.42)^a^	-
	Total	374	100.00	264.67 (69.65)^a^	74.71 (19.66)^a^	-
Total cost						0.00
	Transportation	-	24.78	315.15 (82.93)^a^	239.46 (63.00)^a^	-
	Lodging	-	9.26	117.8 (31.0)^a^	196.56 (51.72)^a^	-
	Food	-	65.95	838.67 (220.70)^a^	470.58 (123.83)^a^	-
	Total	374	100.00	1,271.6 (334.63)^a^	694.56 (182.78)^a^	-
Start time						0.00
	Days	374	-	12.62	7.56	-
Duration of regimen performed						0.00
	Weeks	374	-	4.8	2.2	-
% of regimen performed						0.00
	Total	398	-	89.17	28.62	-
	Received at least one session	374	-	94.36	19.38	-

a Value in American dollars (1 dollar is equal to 3.80 soles)

SIS: Comprehensive Health Insurance, prioritised for vulnerable populations living in poverty and extreme poverty. ESSALUD: Social Health Insurance, for the employed and their families. Or independent workers who are able to make their contributions

* Statistical significance (*p* < 0.05)

**Table 2.** Association and correlation between the independent and dependent variables.

Independent variable	Dependent variable	Test value	*p*-value
Type of insurance^a^	Weekly cost^d^	13,412	0.070
	Start time (days)^d^	13,033	0.028^*^
	Teletherapy performed	16,290	0.196
	Worst treatment outcome (months)^d^	13,245	0.156
Distance^b^	Weekly cost^d^	0.484	0.000^*^
	Weekly transport cost^d^	−0.044	0.393
	Weekly lodging cost^d^	0.798	0.000^*^
	Weekly food cost^d^	0.131	0.011^*^
	SIS-ESSALUD/Private start time (days)^d^	0.036	0.489
	SIS start time (days)^d^	0.129	0.033^*^
	ESSALUD/private start time (days)^d^	−0.580	0.517
	Teletherapy performed	−0.104	0.038^*^
	Worst treatment outcome (months)	−0.239	0.034^*^
Type of neoplasm^c^	Weekly cost^d^	0.857	0.836
	Start time (days)^d^	3.522	0.318
	Teletherapy performed	4.309	0.230
	Worst treatment outcome (months)^d^	3.682	0.196

a Mann–Whitney *U* test

b Spearman’s Rho correlation test

c Kruskal–Wallis test

d Only patients who received at least one teletherapy session were analysed (*N* = 374)

* Statistical significance (*p* < 0.05)
